# Review of Mouse Models of Graves’ Disease and Orbitopathy—Novel Treatment by Induction of Tolerance

**DOI:** 10.1007/s12016-016-8562-7

**Published:** 2016-07-02

**Authors:** Martin Ungerer, Julia Faßbender, Zhongmin Li, Götz Münch, Hans-Peter Holthoff

**Affiliations:** Procorde (Advancecor), Fraunhoferstrasse 9a, 82152 Martinsried, Germany

**Keywords:** Thyreotropin receptor, Graves’ disease, Autoimmunity, Peptides, Tolerance

## Abstract

Various approaches have been used to model human Graves’ disease in mice, including transfected fibroblasts, and plasmid or adenoviral immunisations with the extracellular A subunit of the human thyrotropin receptor (TSHR). Some of these models were only observed for a short time period or were self-limiting. A long-term model for human Graves’ disease was established in mice using continuing immunisations (4-weekly injections) with recombinant adenovirus expressing TSHR. Generation of TSHR binding cAMP-stimulatory antibodies, thyroid enlargement and alterations, elevated serum thyroxin levels, tachycardia and cardiac hypertrophy were maintained for at least 9 months in all Ad-TSHR-immunised mice. Here, we show that these mice suffer from orbitopathy, which was detected by serial orbital sectioning and histomorphometry. Attempts to treat established Graves’ disease in preclinical mouse model studies have included small molecule allosteric antagonists and specific antagonist antibodies which were isolated from hypothyroid patients. In addition, novel peptides have been conceived which mimic the cylindrical loops of the TSHR leucine-rich repeat domain, in order to re-establish tolerance toward the antigen. Here, we show preliminary results that one set of these peptides improves or even cures all signs and symptoms of Graves’ disease in mice after six consecutive monthly injections. First beneficial effects were observed 3–4 months after starting these therapies. In immunologically naïve mice, administration of the peptides did not induce any immune response.

## Background: Clinical Features of Disease

Graves’ disease is a common antibody-mediated autoimmune condition targeting the thyrotropin-TSH receptor (TSHR) in the thyroid gland, resulting in hyperthyroidism [1], with an annual incidence of 15–80 per 100,000 persons throughout the world. A quality of life (QOL) assessment showed that the disease is accompanied by a reduced vital and mental QOL for several years despite treatment according to current standards [2]. If left untreated, Graves’ leads to significantly increased morbidity and mortality [2].

## Animal Models of Disease—Thyroid Alterations and Hyperthyroidism

The antibody-mediated autoimmune condition targeting the TSH receptor (TSHR) in the thyroid gland which results in hyperthyroidism can be replicated in mouse models [3], preferably in TSHR-immunised mice. This was initially observed in the transfected fibroblast model [4], in which about 25 % of injected mice developed hyperthyroidism, and after recombinant dendritic cell injections [5]. After some failed efforts to use plasmid immunisations, adenovirally induced immunisation with the A domain of TSHR (Ad-TSHR) in BALB/c mice was successfully established to induce a mouse model of Graves’ disease [3, 6, 7]. The use of adenovirus was more effective than use of plasmid transfection in most studies, with the exception of a protocol of electroporation [8, 9]—four 3-weekly immunisations over a total period of 3 months. After plasmid electroporation, long-term expression of stimulatory anti-TSHR antibodies and persistent thyroid enlargement was described [10]. However, this disease model seems to have caused a relevant mortality of the investigated mice starting 4 months after the last electroporation [10].

The establishment of various protocols to induce Graves’ disease led to the challenge of comparing these models [3, 7]. Elevated T4 levels, stimulating anti-TSH antibodies, goiter and typical histological alterations, were considered to be essential characteristics of any model which should be relevant for human disease [3, 7], and most were replicated already in early reports [11, 12].

Long-term persistence of the models using adenoviral gene transfer was not clearly described in most of these studies. Prolongation of the protocol based on three adenovirally induced immunisations over 6 weeks and measurements after 20 weeks instead of 10 weeks also led to disease induction [13]. The immunisations with recombinant TSHR were studied in both, wild-type or TSHR-transgenic mice. Although the formation anti-TSHR antibodies with binding activity was consistently observed after 20 weeks in these virus-immunised mice, the potency of the resulting antibodies to stimulate cAMP considerably diminished or even disappeared during the course of the experiment (50 % positive at the end of the experiment), and no T4 elevation nor thyreocyte hyperplasia was observed [13]. Regulatory T cell depletion and administration of adjuvant in TSHR-transgenic mice did not reliably induce Graves’ disease [14].

In a recent study [15], we extended these existing models of adenoviral TSH receptor immunisation by using a novel protocol in which regular injections were continued for 9 months, in order to permanently boost antibody production in mice. This protocol was established in parallel to a previous study which successfully introduced a long-term disease model of cardiomyopathy caused by anti-β1 receptor antibodies in rats [16, 17].

This extended protocol of three 3-weekly immunisations—followed by regular four weekly boosts led to generation of TSH-binding antibodies, which stimulated TSHR-dependent cAMP activity and induced thyroid hyperplasia and marked histological alterations for at least 9 months, as well as consistent thyroxin (T4) release.

## Anti-TSHR Antibody Titres and Capacity to Stimulate cAMP in Test Cells

Anti-TSHR antibodies were determined from serum samples using three different assays. These assays included the current gold standard “third generation” immunoassay, which detects the ability of the respective mouse sera to inhibit the binding of the monoclonal Graves’ patient antibody M22 to the TSHR (RSR-Cobas Roche), which is most often used to identify Graves’ disease in humans. This assay was reported to identify Graves’ patients with a specificity and sensitivity of >97 % [18, 19]. So, functionally active anti-TSHR antibodies observed in immunised mice are readily detected by this same assay. Use of identical assays also allowed to compare absolute titre values. Mean anti-TSHR antibody titres increased progressively during the course of the study in Ad-TSHR-immunised animals, as determined by third generation ELISA.

Very comparable results were obtained with a second-generation assay using competition against the physiological agonist TSH [15].

In addition, the stimulatory activity of these antibodies was determined as the capacity of mouse serum samples to stimulate TSHR-dependent cAMP levels in test cells [15]. This capacity was markedly and consistently increased in serum samples taken from Ad-TSHR-immunised animals but was never detected in sera from the mock-immunised group.

## Sizes and Patho-histological Changes of the Thyroid, Serum Thyroxin Levels

Macroscopic investigation showed clearly increased thyroid sizes in mice which had received nine immunisations of recombinant adenovirus encoding TSHR (Ad-TSHR). Thyroid volumes were determined from the sums of the areas of standardised sections throughout the thyroid. In addition, consistent and marked thyroid hyperplasia was observed. Histological features included increased thyrocyte length and cuboid epitheloid hyperplasia after 7 months, and a degenerate image with prominent infoldings of follicles, smaller follicle size and vacuolisation at 9 months [15]. This led to fractioning of thyroid follicles, and corresponding smaller follicle and colloid sizes [15]. This degenerate histological image contrasted with the normal aspect of intact follicles and normal colloid size of mock-immunised animals.

Mean T4 levels in the group in Ad-TSHR-immunised animals were consistently and significantly higher than controls, if 10^10^ plaque-forming units (pfu) of recombinant Ad-TSHR were used for immunisation [15].

## Graves’ Orbitopathy

Animal models of Graves’ disease have been tested especially for their ability to reproduce orbital histology and to quantify retro-orbital fibrosis which represents an important hallmark of clinical disease in humans. After some prior studies had failed to find such alterations in TSHR-immunised mice, histological investigation after transcranial dissection documented altered orbital muscle fibrosis in single mice after electroporation and plasmid gene transfer of the TSHR A domain [8]. Interestingly, these orbital alterations did not occur in animals which had been transfected with the gene coding for the insulin-like growth factor receptor-1 (IGF1R) which has also been implied in the pathogenesis of Graves’ orbitopathy, although relevant anti-IGF1R antibody titres were documented in these animals. A further elegant study by the same group [9] also documented inflammatory alterations in the orbitae of TSHR-immunised animals histologically. Additional nuclear resonance imaging was used to assess orbital and orbital muscle volumes serially over time and showed clear increases of both parameters [9].

Based on these studies, these authors hypothesised that orbital pathologies do not occur after adenoviral TSHR immunisation, but only after TSHR plasmid immunisation [20]. However, this conclusion was based on the analysis of papers studying short-term adenoviral immunisation protocols.

**Fig. 1:**
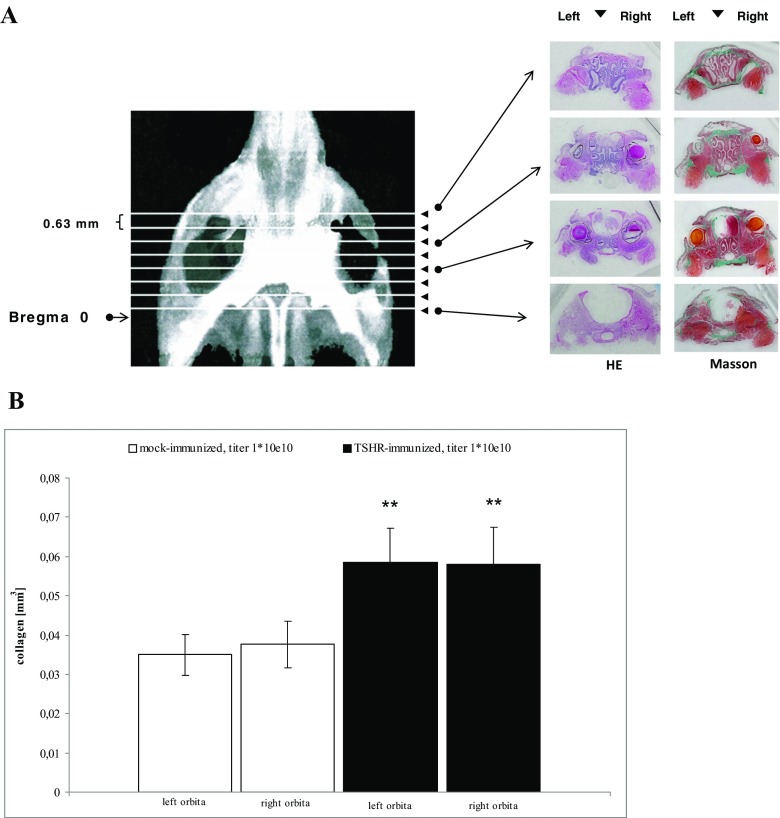
Histological investigation of orbital sections. **a** Representative macroscopic images. Representative images of coronary sections of a mouse orbita and neighbouring tissues. The sections were taken at defined distances from the mouse bregma. Interstitial connective tissue/fibrosis was then stained in green (Masson’s trichrome stain). For clarity, both HE-stained sections (*left panels*) and Masson’s stained sections (*right panels*) are shown next to each other. **b** Effect on digitised analysis of retroorbital tissue. The effects on severity of retro-orbital fibrosis were evaluated in histological sections. The measurements were carried out in immunised mice treated by either four weekly injections with control Ad-GFP (“mock-immunised”) or four weekly injections with Ad-TSHR (“Graves’ diesease”). *N* = 10 mice were investigated in each group. The mean total fibrosis volumes of each right and left orbita, as assessed by digitised image analysis of all sections, and consecutive integrations, are shown with SEM. Differences between groups were tested by ANOVA, *p < 0.05 compared to the TSHR-immunised group

Since we had observed that long-term repeated adenoviral TSHR immunisations seem to induce a more stable Graves’ phenotype in mice [15], we wished to investigate the orbitae in a follow-up study using an identical protocol using nine immunisations with 10^10^ pfu Ad-TSHR. Analysis of orbital changes was carried out histologically using a protocol which included many steps which had already described in the previous studies [8, 9], including Masson’s trichrome staining, but added a few modifications:

For orbital preparations after euthanasia, complete dissections of the orbital and periorbital areas included all orbital tissues, eyelids and adjacent tissues. Consecutively, the tissues were trimmed, fixed and decalcified by placing in EDTA solution for 48 h, then washed 3× with phosphate-buffered saline (PBS). Then, the tissues were immerged into a sucrose solution for 24 h at 4 °C, followed by fine-trimming and incubating in optimum cutting temperature (OCT) formulation. Serial coronary sections (7 μm thick, 0.63 mm apart) were obtained (Fig. 1a). For Masson’s staining, sections were placed in Bouin’s fixation solution at 20 °C overnight and washed for 2 h, and then treated with Masson-Goldner trichrome staining kits.

The orbital sections were captured with an Axiovision digital cam system. Fibrosis areas in the extra-orbital adipose tissues and extra-orbital muscle (EOM) regions were indicated by their green colour after Masson’s trichrome staining. Digitised image analysis of the green colour pixels areas enabled reliable quantification of fibrosis, as had been validated previously [21]. Accordingly, all fibrotic tissue throughout a whole orbital section was quantified, and results of all sections were added in the end to yield a total fibrosis volume (mm^3^) of each investigated orbita.

As an extension of our previous results [15], we now show in a follow-up study that a significant increase of retro-orbital fibrosis is observed in mice if adenoviral TSHR immunisations are maintained using the identical protocol for several months. Figure 1b shows that this increase occurs in both, right and left orbitae. These findings complement the previous studies in which orbital pathology was assessed by magnetic resonance (nuclear spin) imaging MRI [9].

## Cardiac Involvement in Patients

A clinical feature of all forms of hyperthyroidism is cardiac involvement which is directly caused by the action of thyroid hormones on nuclear receptors within the myocardium [22]. Hence, contractility is increased, and a hypertrophic form of cardiomyopathy [23], as well as tachycardia is observed in these patients. Heart rates at rest are increased and represent a reliable marker of disease severity [22, 23]. Atrial fibrillation occurs in 5–15 % of patients [24], and palpitations are among the most reported symptoms defining disease burden. Increased cardiovascular morbidity and mortality have been reported in patients with both, overt or subclinical hyperthyroidism [24–27]. Hyperthyroid patients suffering from Graves’ disease are at especially increased cardiovascular risk [28]. Graves’ disease results in increased morbidity and mortality [2] mainly due to cardiac complications [22]. Twenty-four-hour ECG monitoring showed that heart rate is constantly increased during the day [23].

## Cardiac Involvement in Animal Disease Models of Graves’ Disease

Effects of Ad-TSHR immunisation were reported in three rhesus macaque monkeys which were successfully treated with seven consecutive injections over 20 weeks [29]. Clinical investigation of these three animals also indicated increased heart rate (no details on method were given). In a previous study, we showed that also in Ad-TSHR-immunised hyperthyroid mice, significant tachycardia occurred [15]. Most of the evaluated ECGs related to sinus tachycardia—short stretches of arrhythmia occurred, but could not be unequivocally related to atrial fibrillation, perhaps owing to registration quality.

Macroscopical investigation and preparation of mouse hearts upon necropsy revealed significantly increased heart weights after Ad-TSHR immunisation [15]. Additionally, calculation of myocardial volumes by adding up digitised cross-sectional LV areas which were calculated from standardised heart sections showed increased cardiac ventricular volumes.

Histological assessment revealed signs of cardiomyocyte hypertrophy and cardiomyopathy. These included increases in cell size and thicker myocardiac fibres in the HE-stained sections of the Ad-TSHR-immunised animals.

Table 1 shows an overview on mouse models of Graves’ disease especially with regard to cardiac and orbital involvement.

**Table 1:** Overview on major mouse models of Graves’ disease with a focus on cardiac involvement and orbitopathy

Approach	Hyperthyroidism	Cardiac changes	Orbitopathy	Reference
Cells which stably express TSHR				
Transfected fibroblast model	About 25 %	n/d	n/d	[4]
Recombinant dendritic cells	Low incidence	n/d	n/d	[5]
TSHR DNA (plasmid) administration				
Intramuscular injection	17 %	n/d	Yes	[69]
Intramuscular injection w/ G2 plasmid	None	n/d	Yes	[70]
Electroporation, follow-up 3 months	High incidence	n/d	Yes	[8, 9, 20]
Electroporation, follow-up 9 months	High incidence	n/d	n/d	[10]
Adenovirus encoding TSHR (A domain)				
Up to three immunisations over 4 months	25–70 %	n/d	No	[3, 6, 7, 11, 12]
Up to three immunisations, follow-up 20 weeks	No/low incidence	n/d	No	[13]
Nine immunisations over 9 months	High incidence	Yes	Yes	[15]
				+ Data shown here

*n/d* not determined/not reported

## Correlations

Correlations were determined for end-of-study results in TSHR-immunised mice. A study on plasmid-induced TSHR immunisation found reasonable correlations between thyroxin T4 value and anti-TSHR antibody titres [10]. Also, after Ad-TSHR immunisation, we found high correlations coefficients >0.7 for thyroid sizes vs. thyroxin (T4) levels, vs. anti-TSHR titre levels and vs. heart weights and heart rates at the end of the observation period [15]. In contrast, thyroid sizes did not correlate as well with the absolute cAMP-stimulatory capacities of the anti-TSHR antibodies on test cells, which were generally not well correlated to the other parameters.

These findings indicate that the animal model is consistently characterised by generation of specific anti-TSHR antibodies whose levels in single animals impact on the amount of thyroid enlargement and on serum T4 levels as well as on the cardiac consequences of disease. We also conclude from these data that effects of anti-TSHR antibodies on target cells different from cAMP stimulation should be relevant, because the biological effect of these antibodies is not precisely predicted just by measuring their capacity to stimulate cAMP levels in test cells. Such alternative second messenger systems could depend on stimulation of Gq and consequent activation of phospholipase C or other intracellular enzymes but might also imply activation of Gi-dependent pathways.

## Therapeutic Approaches

Graves’ disease is often initially treated by giving thyreostatic drugs, such as carbimazol, followed by radioiodine therapy [30] or surgical removal of the thyroid gland. All these treatment options are characterised by relatively high relapse rates and significant side effect profiles [31].

Treatment of refractory disease cases and of accompanying ophthalmopathy/orbitopathy is especially challenging. Ophthalmopathy occurs in almost half of all Graves’ patients—up to 16 per 100,000 women per year in the general population [32]. In this condition, anti-TSHR antibody titres and relapse rates are especially high [32]. These patients must frequently be treated with high doses of intravenous corticoids over many weeks, which even incur more side effects [33]. Therefore, novel treatment options have been explored in recent years. A reduction of B lymphocytic cell counts can be achieved by giving the anti-CD20 antibody rituximab (MabThera^®^, anti-CD20 Mab). Driven by the hypothesis that Graves’ disease is majorly a B cell-mediated condition, a recent randomised double-blind trial showed an advantage for the rituximab-treated group [34], whereas another did not (NCT 00595335, ref. 33), perhaps due to frequent side effects of the therapy.

## Recent Novel Experimental Therapies Which Were Investigated in Mouse Disease Models

Studies in Ad-TSHR-immunised mice established that the model can be used to investigate immunotherapeutic interventions: a mouse anti-CD20 antibody, an analogue of rituximab [35] and the B cell activating factor fusion protein TACI-Fc (atacicept, ref. [36]), which can block the B cell activating factor BAFF (also known as B lymphocyte stimulator, BLys) on these cells. Many of these rather broad-range B lymphocyte-directed therapies have been successfully established in the treatment of a variety of autoimmune disorders and reflect the increasing recognition that not only regulatory T lymphocytes but also B lymphocytes play an important role in autoimmunity [37, 38].

Further work at FIRS lab, RSR, Cardiff, has identified inhibitory monoclonal TSHR-binding antibodies such as K1-70 which were isolated from patient blood and which compete for the stimulatory action of Graves’ patients’ autoantibodies [39]. They can counteract hyperthyroid states in M22-injected rats [39].

In addition, small molecule TSHR antagonists were conceived, but so far, only tested ex vivo and in healthy mice [40–42]. These compounds such as ANTAG3 are specific allosteric regulators of the TSHR hinge region but seem to act only at fairly high in vivo doses. In addition, all suffer from some cross-reactivity with the luteinising or follicle-stimulating (LH or FSH) hormone receptors at least at the high doses which are needed for in vivo effects, and their toxicological characterisations have so far not been reported.

## Prospect of Allergen-Specific Immune Therapies in Patients

In general, treatment with broad-range immunosuppressive drugs may cause serious side effects so that, in addition, antigen-specific therapies have been conceived to induce tolerance in a variety of autoimmune conditions. For many decades, specific immune therapies (SIT) have been established successfully for the treatment of allergic-atopic diseases. Recombinant peptides are being increasingly used for such hyposensitisation therapies (SIT) which offer significant advantages over the classical raw allergen extracts [43–45].

## Effects on T Lymphocytes

In analogy to SIT for allergic diseases, peptides to induce tolerance have been investigated in a variety of other conditions and been linked to regulatory T cells. Generally, specific immunotherapy for autoimmune diseases lagged behind until the discovery that T helper lymphocytes are activated by peptides bound to major histocompatibility (MHC) class II proteins [46]. This led to the design of peptides that selectively target immune cells without risking the activation of self-reactive cytotoxic T or B lymphocytes [43]. Exposure to a peptide which stimulates self-reactive T helper lymphocytes can mitigate the disease, which may be explained by the “two-signal” rule of T cell activation [46, 47]: Self or foreign antigens must be broken down into peptides, which bind MHC class II proteins and must be displayed at the surface of antigen-presenting cells (APCs) to activate effector cells. This step is known as signal 1. The antigen-presenting cells must also upregulate costimulatory molecules, such as CD80 and CD86, to provide the second signal required for T helper cell survival and proliferation.

At conditions when T helper cells receive signal 1, but not signal 2, they induce a state of unresponsiveness known as anergy [48]. In a recent publication, T helper cells treated with nanoparticles which had been coated with a peptide bound to MHC class II proteins (pMHC-NP treatment) triggered signal 1 alone [49, 50]. This concept has been shown to not only induce anergy but also drive T helper cells to differentiation to what resembles regulatory T cells which reduce immune responses [49, 50].

Altered regulation of immune checkpoint interaction between CD 28 and CD80/86 and CTLA-4 has been implied in these processes [51]. If applied subcutaneously, successful therapy has been related to consecutive antigen dose escalation and to local release of interleukin-10 from lymphocytes [52, 53]. Although immune-disease-triggered epitope spreading might have occurred at the time when such a therapy is initiated [54], “bystander suppression” of independent epitopes has been implied in therapeutic effects [53]. Although these therapies have not all been successful, they are still being pursued by researchers in various fields [55, 56].

## Novel Approach for Antigen-Specific Therapy: Intravenous Administration of High Doses of Conformational Peptides

As a novel option for antigen-specific therapy, the group of Martin Lohse and Roland Jahns has established intravenous administration of fairly high doses of immunogen-mimicking cyclic peptides for the treatment of anti-β1-adrenergic receptor-mediated autoimmune cardiomyopathy [16, 57–59]. The investigation of peptide-treated animals showed that the beneficial therapeutic effects were related to a marked decrease of antigen-specific pre-B lymphocytes in the spleen [57]. This phenomenon can be observed if these cells are exposed to the specific antigen in the absence of a co-stimulatory signal, which again refers to the lack of interaction between CD80/86–B7.1/2 and CD28.

**Fig. 2:**
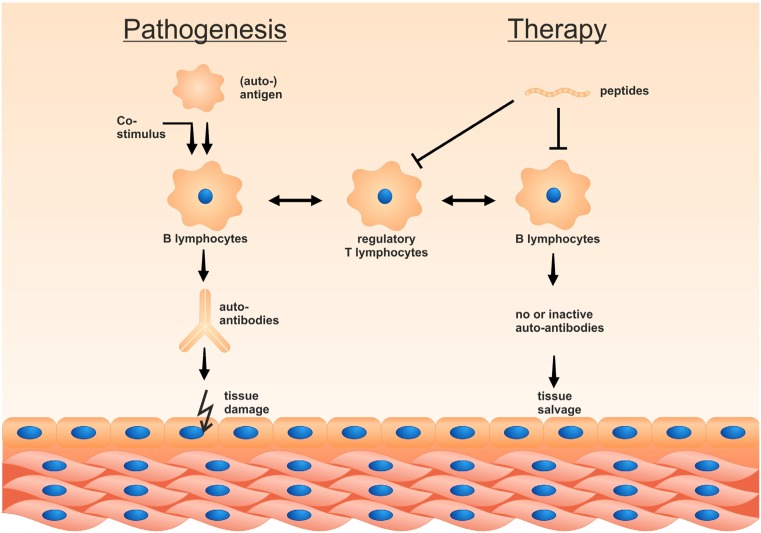
Antigen-specific peptides can induce peripheral tolerance. The figure shows that administration of peptides can induce peripheral T or B lymphocyte anergy if presented to antigen-presenting immune cells in the absence of a co-stimulatory signal

Figure 2 very schematically shows the possible modes of action of such peptides, i.e. to induce peripheral tolerance. Tolerogenic peptides can reduce immunogenic T lymphocytes and induce specific subsets of tolerogenic (regulatory) T lymphocytes. Simultaneously/alternatively, they can reduce antibody-producing lymphocytes and pre-B lymphocytes by anergy. Recent studies have implied induction of regulatory T helper subsets instead of T lymphocyte anergy during this process [46], documenting such changes not only in rodents but also in human cells.

## Effects on B Lymphocytes

In parallel to T lymphocyte anergy, B cell anergy has been described in several pivotal studies in which it was shown to require constant B cell receptor (BCR) occupancy with rather high levels of (self) antigen [60, 61]. After initial characterisation in rodents, it has also been reconfirmed in human immune cells [62, 63] and been explained by chronic low-level BCR cross-linkage [63]. In addition, high-level BCR cross-linkage in the absence of co-stimulatory signals leads to clonal B cell deletion after acute high level antigen exposure [63]. Originally, clonal anergy was proposed as a way to inactivate B cells stimulated early in development when only autoantigens would be presented [60]. Further studies have documented fine tuning of B lymphocyte responsiveness and inactivation of self-reactive B cell clones by anergy [64]. This phenomenon seems to play a relevant role in a variety of immune phenomena and seems to be an attractive mechanism for the treatment of several B lymphocyte-mediated diseases. For more than a decade, the direct effects of B lymphocytes in autoimmune disease have been increasingly recognised. Their importance is highlighted by the fact that auto-antibodies can often be detected in these diseases many years before symptoms evolve (i.e. in thyroid autoimmune diseases) and that disease manifestations can be transferred across the placenta, which should be due to the effect of pathogenic maternal antibodies on embryos [37].

Similar to what has been described for T lymphocytes, antigen-mimicking peptides may be presented to these immune cells via major histocompatibility class II (MHC II)-dependent antigen presenting cells in the absence of co-stimulatory signals and thereby reduce their activation.

## Antigen-Specific Immunotherapy for Thyroid Disease in Animal Models

For several years, studies in Ad-TSHR-immunised mice have been used to investigate antigen-specific immune therapies as novel interventions: An early specific immune therapy approach in mouse models of Graves’ disease relied on intranasal administration of linear peptides as T cell epitopes which however did not prove successful [65]. Alternatively, a soluble form of the TSHR A domain (expressed as his-tagged protein in CHO cells) was shown to induce tolerance in mice when given before subsequent Ad-TSHR immunisations [66]. However, a single intra-muscular or subcutaneous injection of this protein was not effective on established disease in this short time model of Ad-TSHR immunisations: Anti-TSHR titres did not decrease, but rather increased, and T4 thyroxin levels were unaltered in these mice.

In another study, intra-peritoneal or intra-muscular challenges of Ad-TSHR on neonatal mice had induced tolerance against further immunisations in adulthood [67]. However, such a virus-mediated prophylaxis may be difficult to establish in human medicine.

Last not least, another approach identified peptides to induce tolerance after selecting specific T cell epitopes which were optimised in HLA-DR 2-transgenic mice. Subcutaneous administration of the peptide ATX-GD-5D-K has been shown to reduce anti-TSHR antibody production after consecutive adenoviral TSHR immunisation in a similar mouse model [68], i.e. in a prophylactic setting. The concept is that soluble peptides bind to empty MHC receptors and selectively trigger activation of interleukin-10-positive T lymphocytes which specifically suppress pathogenic T helper cells [68]. The authors have so far not published data on treatment of established disease nor on thyroid size or hyperthyroidism.

## Intravenous Conformational Epitope Peptide Therapy for Thyroid Disease

**Fig. 3:**
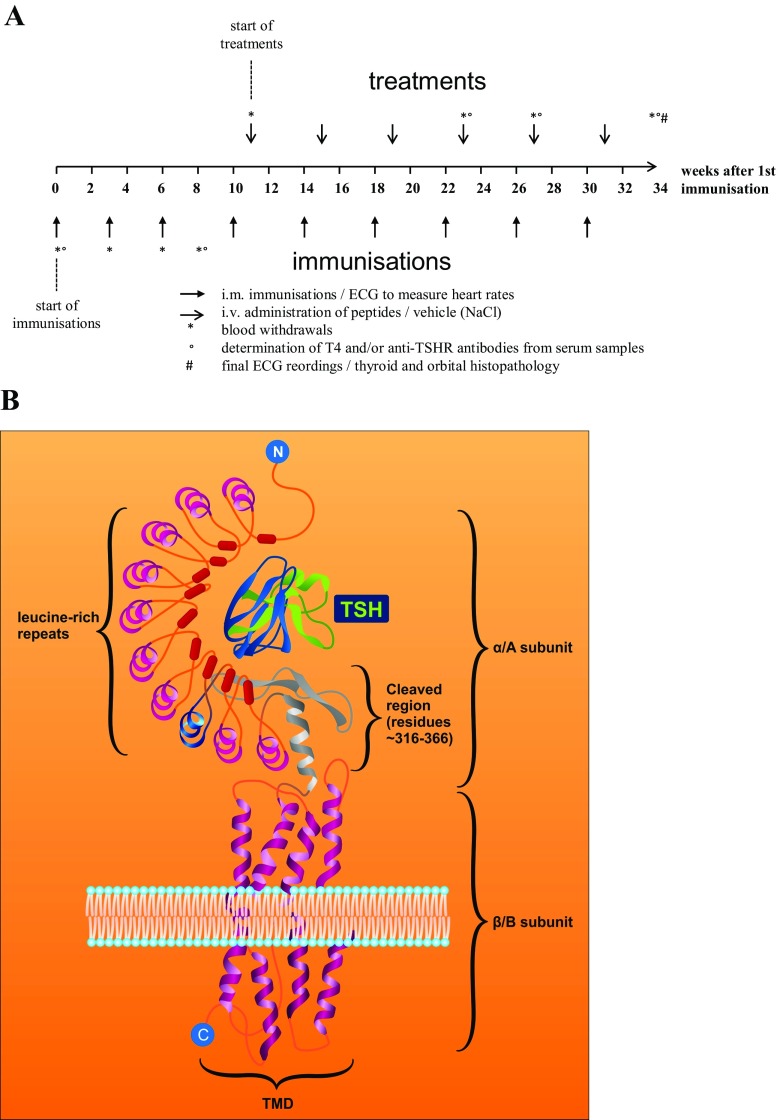
**a** Time schedule of the currently on-going therapeutic study. The figure shows the time course of immunisations, of therapeutic peptide administrations and of measurements. **b** Schematic structure of the thyroid stimulating hormone (*TSH*) receptor. Peptides were derived from the loop structure of the leucine-rich repeat domain of the extracellular A subunit of the TSHR, as marked in *blue colour*

In parallel and as an extension to these approaches, we sought to establish another antigen-specific therapeutic concept for anti-TSHR-mediated disease. In parallel to the studies on β adrenergic receptors [57], we designed peptides which mimic the tertiary structure of the single cylindrical loops of the TSHR leucine-rich repeat domain (LRD). TSHR and β-adrenergic receptors share some common features since they are both G protein-coupled receptors. Figure 3b shows the structure of the LRD and indicated how novel peptides were derived from the native structure of the receptor.

These peptides were tested for their potency to induce tolerance in TSHR-immunised diseased mice after repeated intravenous administration of target doses—to mitigate disease manifestations such as thyroid enlargement, hyperthyroidism and retro-orbital fibrosis. To this end, an identical protocol was used as described before [15], and complemented by therapeutic peptide administrations after week 11, when thyroid disease had fully evolved. All peptides were administered six times at four weekly intervals. Please see Fig. 3a for an overview on the protocol of immunisations and of therapeutic peptide administrations.

**Fig. 4:**
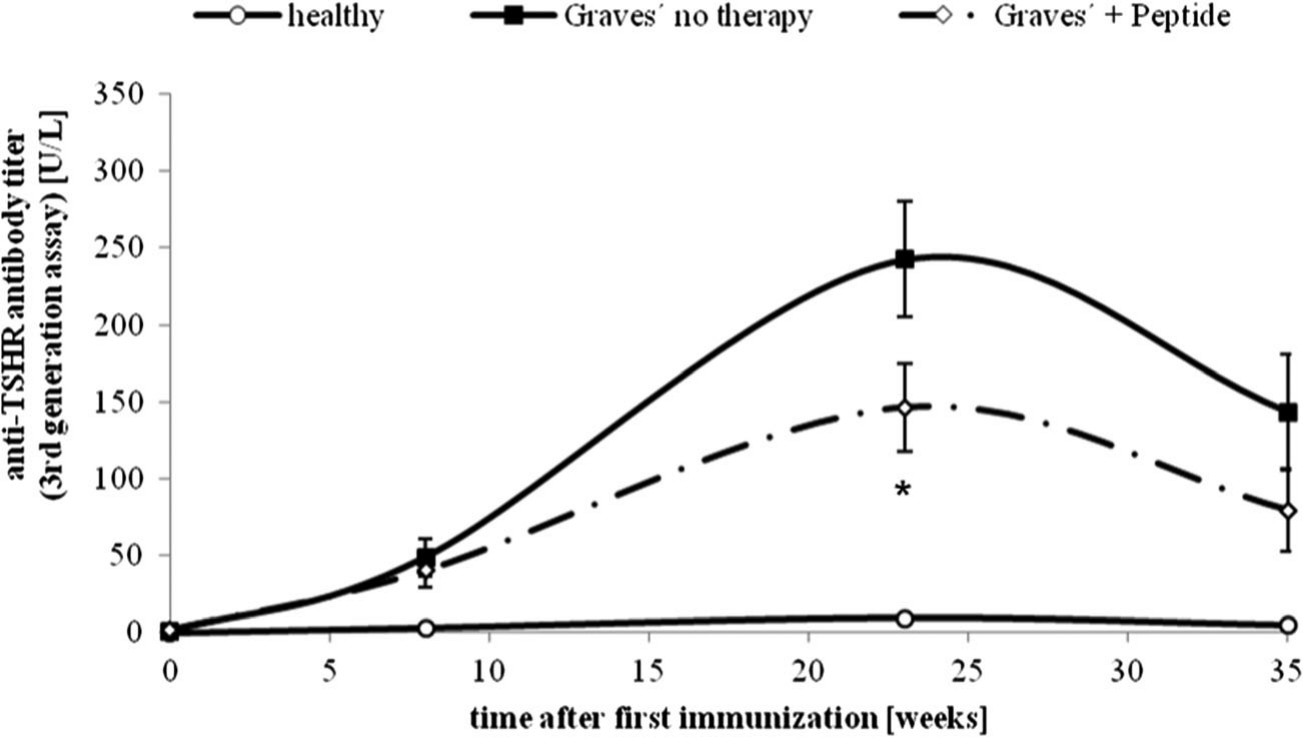
Effect of novel peptides on anti-TSHR antibody titres. The effect of peptide therapy on time course of anti-TSHR titres, as measured by third generation ELISA. The measurements were carried out in Ad-TSHR-immunised mice treated by either four weekly injections with vehicle (“Graves’—no therapy”), or administrations of a peptide (1 mg/kg body weight—“Graves’ + peptide”). In addition, age-matched immunologically naïve mice were investigated (“healthy”). *N* = 10 mice were investigated in each group. Data are represented as mean ± SEM. Significance over time was tested by analysis of variance (ANOVA) of groups at given time points and controlled by ANOVA for repeated measurements within one group, followed by LSD post hoc testing. *Asterisk* indicates statistical significance (*p* < 0.05) compared to the TSHR-immunised group treated with only NaCl

Our preliminary experiments show that a set of peptides which mimic a loop of the leucine-rich repeat domain of TSHR suppressed or at least stabilised the titres of TSH-binding antibodies despite continuing immunisations (Fig. 4).

**Fig. 5:**
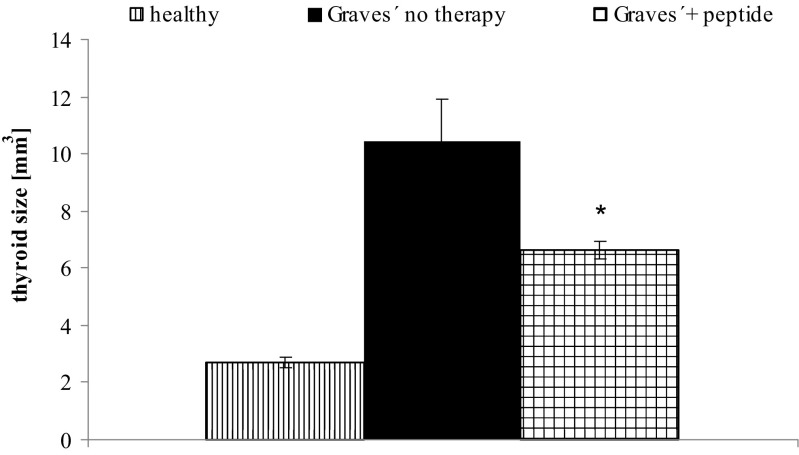
Effect of peptides on macroscopically measured thyroid size. The effects of peptide therapy on thyroid sizes were investigated at the end of the experiment. The measurements were carried out in Ad-TSHR-immunised mice treated by either four weekly injections with vehicle (“Graves’—no therapy”) or administrations of peptide (1 mg/kg body weight). In addition, age-matched immunologically naïve mice were investigated. *N* = 10 mice were investigated in each group. The mean thyroid sizes in mm^3^ are shown with SEM. Differences between groups were tested by AVOVA followed by post hoc LSD testing. *Asterisk* indicates statistical significance (*p* < 0.05) compared to the TSHR-immunised group treated with only NaCl

**Fig. 6:**
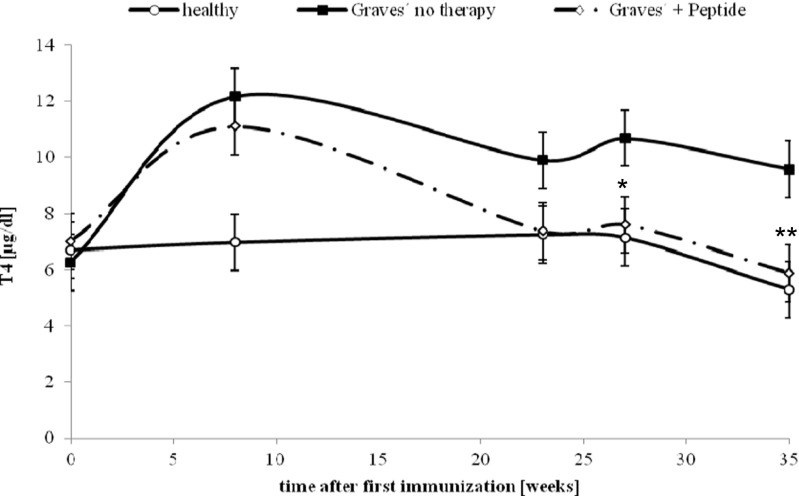
Effect on serum thyroxin (T4) levels. The effects of peptide therapy on serum thyroxin levels were evaluated. The measurements were carried out in Ad-TSHR-immunised mice treated by either four weekly injections with vehicle (“Graves’—no therapy”) or of a peptide (1 mg/kg body weight), or in age-matched immunologically naïve mice. Data are represented as means ± SEM. *N* = 10 mice were investigated in each group. Significance over time was tested by analysis of variance (ANOVA) of groups at given time points and controlled by ANOVA for repeated measurements within one group, followed by LSD post hoc testing. *p < 0.05 and **p < 0.01, compared to the TSHR-immunised group treated with only NaCl

Thus, established thyroid disease was successfully treated in these animals. To our knowledge, this is the first report on such findings after antigen-specific therapy. Increased thyroid sizes were reduced after 6 months of peptide therapy (Fig. 5). In parallel, also thyroid hyperplasia and histological alterations were markedly reduced. Elevated thyroxin (T4) levels were reverted to normal values, starting 15 weeks after initiation of peptide therapy (Fig. 6).

**Fig. 7:**
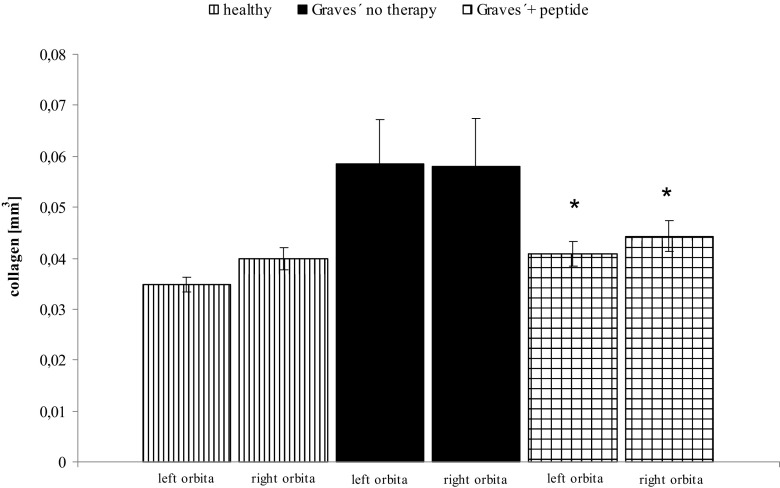
Histological investigation of orbital sections and digitised analysis of retroorbital tissue: effects of novel peptides. The effects of peptide therapy on severity of retro-orbital fibrosis were evaluated in histological sections. The measurements were carried out in mice which had either received four weekly injections with vehicle (“Graves’—no therapy”) or of a peptide (1 mg/kg body weight), or in age-matched immunologically naïve mice. The mean total fibrosis volumes of each right and left orbita, as assessed by digitised image analysis of all sections, and consecutive integrations, are shown with SEM. *N* = 10 mice were investigated in each group. Differences between groups were tested by ANOVA, *p < 0.05 compared to the TSHR-immunised group treated with only NaCl. Also, results of TSHR-immunised mice differed significantly (*p* < 0.05) from healthy controls

Initial results of a follow-up study also suggest that retro-orbital fibrosis was reduced, suggesting a positive effect on Graves’ orbitopathy (Fig. 7). These findings represent an interesting complementation of previous preclinical studies on Graves’ disease models, since such a therapeutic effect has not yet been shown in animal models. On the other hand, patients with Graves’ orbitopathy are especially hard to treat.

Since the investigated peptides were derived from only one of the several possible epitopes of which the LRD of the TSHR is composed, the feasibility to block polyclonal immune responses in different mice suggests that “linked suppression” (sometimes also termed bystander suppression) plays a role in the observed effects, although so far only described for T lymphocyte-directed therapies.

In addition, administration of the same peptides at identical dosings (six monthly administrations) in immunogically naïve mice did not result in any immune reactions. No anti-TSHR titres (as measured with the third-generation assay) were observed in these animals at any time during the experiment.

## Summary and Conclusions

In summary, a long-term disease model of Graves’ disease has been established, which stably features many hallmarks of human disease including orbitopathy. Specific peptides which mimic loops of the leucine-rich domain hold promise as a future treatment of Graves’ disease. After repeated monthly administrations, they reduced thyroid size, elevated serum T4 levels, tachycardia and retro-orbital fibrosis. In peptide-treated animals, the titres of TSHR-binding antibodies were suppressed or at least stabilised despite continuing immunisations.

So, current therapeutic approaches to treat autoimmune diseases include administration of increasing doses of allergen-mimicking peptides subcutaneously [53] or of high doses of conformational allergenic epitopes intravenously, which seems to primarily induce B cell anergy [57]. The mechanisms which may explain this immunological tolerance to target antigens remain to be exactly determined—they include B lymphocyte and/or T lymphocyte anergy and regulation of T helper cell populations, as well as clonal B cell deletion.
